# Efficacy and safety of Obex® in overweight and obese subjects: a randomised, double-blind, placebo-controlled clinical trial

**DOI:** 10.1186/s12906-023-03847-7

**Published:** 2023-02-20

**Authors:** Eduardo Cabrera-Rode, Ileana Cubas-Dueñas, Janet Rodríguez Acosta, Jeddú Cruz Hernández, Ana Ibis Conesa González, Teresa M. González Calero, Yuri Arnold Domínguez, José Hernández Rodríguez, Antonio D. Reyes Rodríguez, Aimee Álvarez Álvarez, Ragmila Echevarría Valdés, Liudmila Jorge Espinosa, Onelia Torres Belent, Zoila Bell Benavides, Elizabeth Senra Estévez, Yanet Abreu Rodríguez, Juana del Valle Rodríguez, Silvia Marín Juliá

**Affiliations:** Institute of Endocrinology, University of Medical Sciences of Havana, Zapata and D, Vedado 10400, Havana, Cuba

**Keywords:** Dietary supplementation, Overweight, Obesity, Weight loss, Insulin resistance, Uric acid

## Abstract

**Background:**

Obex® may be helpful in reducing body weight and fat. The current study was carried out to evaluate the efficacy and safety of Obex® in the treatment of overweight and obese subjects.

**Methods:**

A double-blind, randomised, controlled phase III clinical trial was conducted involving 160 overweight and obese subjects (BMI ≥ 25.0 and < 40 kg/m^2^) aged 20 to 60 years, who received Obex® (*n* = 80) and placebo (*n* = 80) plus non-pharmacological treatment (physical activity and nutritional counseling). One sachet of Obex® or placebo were administered before the two main meals each day for 6 months. In addition to anthropometric measurements and blood pressure, fasting plasma and 2 h glucose levels during the oral glucose tolerance test, lipid profile, insulin, liver enzymes, creatinine, and uric acid (UA) were determined, insulin resistance (HOMA-IR) beta-cell function (HOMA-β) were assessed and insulin sensitivity (IS) was calculated with three indirect indexes.

**Results:**

After 3 months of Obex®, 48.3% of the participants (28/58) achieved complete success in reducing both weight and waist circumference by greater than or equal to 5% from baseline, as opposed to 26.0% (13/50) of individuals receiving placebo (*p* = 0.022). Compared to baseline, at 6 months no differences were found between the groups concerning anthropometric and biochemical measurements, except for high-density lipoprotein cholesterol (HDL-c) levels, which were higher in subjects receiving Obex® compared to those receiving placebo (*p* = 0.030). After 6 months of treatment, both groups showed reduced cholesterol and triglyceride levels (*p* < 0.012) compared to baseline value. However, only those intake Obex® showed reduced insulin concentrations and HOMA-IR, improved IS (*p* < 0.05), and decreased creatinine and UA levels (*p* < 0.005).

**Conclusions:**

The consumption of Obex® together with lifestyle changes increased HDL-c, contributed to a rapid reduction of weight and waist circumference, as well as improved insulin homeostasis, which did not occur in the placebo group, and appears to be safe as an adjunct at conventional obesity treatment.

**Trial registration:**

Clinical trial protocol was registered in the Cuban public registry of clinical trials under code RPCEC00000267 on 17/04/2018 and also registered in the international registry of clinical trials, ClinicalTrials.gov, under code: NCT03541005 on 30/05/2018.

## Introduction

The incidence of overweight and obesity has reached epidemic levels and represents a major serious threat to public health on a global scale [1, 2]. The risk of cardiovascular disease, type 2 diabetes (T2D), stroke, high blood pressure, asthma, liver, pulmonary disorders, gall bladder and kidney disease, osteoarthritis, reproductive complications, certain types of cancer, premature death, and a long list of other conditions are all increased by overweight, as determined by body mass index (BMI) [3–7].

Obesity is a major risk factor for T2D and prediabetes [8]. Recent studies estabished that obesity, prediabetes, and insulin resistance are closely associated [9]. Central adiposity distribution is an important risk factor for chronic non-communicable diseases and is independent of excess body weight. The vascular and metabolic complications commonly associated with abdominal or central obesity [waist circumference (WC) and waist-to-hip ratio (WHR)] are a frequent way that comorbidity manifests itself [10–13].

In the United States of America, the age-adjusted prevalence of overall obesity increased from 35.4% in 2011–2012 to 43.4% in 2017–2018 [14].In Cuba, the prevalence of overweight and obesity has risen from 35.5% in 1982 [15] to 56.4% in 2019 (personal communication, Patricia Varona, Cuban National Institute of Hygiene and Epidemiology, National Health Survey, Cuba), which represents a 0.77% linear annual increase.

Obesity has increased globally and at least 2.8 million people die each year from obesity or being overweight. In 2015, a total of 107.7 million children and 603.7 million adults were obese. In more than 70 countries, the prevalence of obesity has doubled since 1980, and it has increased continuously in the majority of other countries [16].

The current explosion in T2D and obesity prevalence rates is a result of significant secular changes in lifestyle and environment which may have a tendency to cluster within families (obvious examples include foetal environment, socioeconomic status, dietary preferences, food availability, a more sedentary way of life, stress, pharmaceutical or endocrine disruptors, and a lower microbial diversity of the gut microbiota composition in obesity) [8, 15–18].

Over the next twenty years, overweight and obesity in all Latin American countries are estimated to increase [19]. Reflecting these trends, the incidence of each disease is also set to increase [19, 20]. In the absence of an obesity intervention provision, obesity will continue to increase in much of Latin America [18, 19]. Nonetheless, several intervention trials have shown favorable effects of weight loss on cardiovascular risk factors and T2D [6, 8, 21–24]. Also, in 2017, a group of researchers published that WC and waist-to-height ratio was more strongly associated with the incidence and prediction of diabetes than BMI [25].

For the treatment of obesity and prediabetes, a number of medicantions have been used, including sibutramine, rimonabant, orlistat, lorcaserin, phentermine/topiramate, exenatide, liraglutide, thiazolidinediones, acarbose, and metformin, among others [22–24, 26, 27]. However, despite the emergence of some promising weight-loss drugs aimed at halting the progression of prediabetes and diabetes, post-marketing surveillance has unfortunately revealed many of these medicatons to be associated with adverse events [23, 24, 27].

The nutritional supplement Obex®, produced by Laboratorios Catalysis, has recently entered the market. It is specifically produced with natural antioxidants and helps to lose weight, with no adverse events having been reported so far. Several articles [28–49] describe the beneficial effect of some specific components of the Obex® supplement on weight loss (Acai berry; helps control adipogenesis by inhibiting the differentiation of pre-adipocytes [28, 29]), waist circumference reduction (inulin, Acai berry, *Caralluma fimbriata;* help control inflammatory markers and promote fat and carbohydrate oxidation [28–36]), appetite suppression (Acai berry, *Caralluma fimbriata*; help control leptin hormone level and satiety receptor sensitivity [31–35]), lowering fasting glucose levels (Acai berry, carnitine, zinc, phasolamine, folic acid, inulin; help fat and carbohydrate metabolism [28–42]), improving insulin sensitivity and β-cell function (L-methionine, L-arginine, L-carnitine, folic acid, zinc; help control oxidative stress and regulate pancreas function [37, 40–45]).

In 2015, Medialdea et al. found that taking the Obex® supplement was beneficial for the treatment of central obesity and prevention of metabolic syndrome in climacteric women. [46] Another report, from an animal study, shows that treatment with Obex® contributed to weight loss, attributed to a reduction in adipose tissue, and it also has a protective effect against an obesogenic environment [47].

In an earlier exploratory study in overweight or obese subjects with impaired fasting glucose levels, consumption of Obex® achieved a reduction in several anthropometric measurements (body weight, BMI, waist circumference, waist-to-hip ratio, and conicity index) and improved fasting glucose levels and high-density lipoprotein cholesterol (HDL-c) concentrations, as well as insulin sensitivity and insulin resistance indexes [48]. Obex® would therefore be a promising supplement to reduce weight and central obesity, as well as to reduce the risk of T2D and cardiovascular disease (CVD).

The aim of this study was to examine the efficacy and safety of Obex® treatment in combination with lifestyle changes on anthropometric, clinical, and biochemical characteristics in overweight and obese individuals.

## Material and methods

### Participants

Subjects were recruited through written advertisements contained in brochures produced for the study. The clinical trial was also promoted through direct communication and via opportunistic population screening in overweight and obese subjects.

Participants were included if they were aged 20–60 years, had a BMI ≥ 25.0 kg/m^2^ and < 40 kg/m^2^ without diabetes mellitus, and agreed to all study visits and procedures.

Participants with any of the most significant systemic, inflammatory or chronic diseases were excluded that were likely to affect study endpoints, as well as a history of or current condition of neurological or psychological disease, including eating disorders or substance use disorders. Participants who took oral or injectable contraceptives and women who were pregnant or breastfeeding were also excluded. Likewise, those who were shown to have thyroid dysfunction (hypo- or hyperthyroidism), type 1 diabetes, type 2 diabetes or prediabetes treated with oral hypoglycaemic agents, as well as those with any of the following characteristics: diseases manifesting insulin resistance (IR) (e.g. acromegaly and endogenous hypercortisolism), sepsis, use of medications that affect weight in the last 3 months, inability to follow instructions, symptomatic hypoglycaemia in the last month, known hypersensitivity to any of the formulation components, use of immunosuppressive drugs in the previous 5 years or having abused alcohol/drugs, were also excluded from the study. Individuals who used steroids, anti-inflammatory medications, multivitamins and/or antioxidants were not allowed.

Patients who did not complete the minimum treatment time (three months) were also excluded. In the case of subjects who completed treatment for at least three months but subsequently discontinued treatment, only the three-month period in which the clinical trial groups were compared was taken into account for analysis.

### Sample size estimation

The estimation of the sample size was based on the rate of weight reduction with non-pharmacological treatment (diet and physical activity) is approximately 15% (p1) and it is assumed that we want to achieve a success rate of 35% (p2) in the experimental group (patients with Obex® treatment), i.e. we aim to detect a difference of 20% between the two treatments, with a Type I error = 0.05 and a Type II error = 0.2. Under the above assumptions, a total of 146 patients would need to be included, 73 in each treatment group. Assuming a probable subject loss of 10.0% during the trial, a total of 160 patients (80 in each treatment group) would then need to be recruited to ensure adequate power.

### Ethical considerations

The study protocol was approved by the Research and Ethics Committee of the Cuban Endocrinology Institute and was conducted following the Declaration of Helsinki. Obex® is registered as a nutritional supplement with the National Institute of Nutrition and Food Hygiene in Havana, Cuba. Written consent was obtained from all patients before the study enrolment. This clinical trial was registered in the Cuban public registry of clinical trials under the code **RPCEC00000267** on 17/04/2018 and in the international registry of clinical trials (ClinicalTrials.gov) under the code **NCT03541005** on 30/05/2018. The Clínical study was approved by the ethics committee of the Institute of Endocrinology, Havana (INEN), on 29/01/2018.

### Study design and dietary supplementation regimen

The present study was a randomised double-blind parallel-group placebo-controlled phase III trial performed at the Institute of Endocrinology, Havana, Cuba, in overweight and obese adults who met the selection criteria.

After an initial evaluation, all subjects who met the eligibility criteria and wanted to participate in the study were enrolled consecutively. The study included 160 subjects who were administered Obex® or placebo (4 g sachets) twice daily for 6 months (24 weeks). In addition, non-pharmacological treatment consisting of lifestyle changes (physical activity and hygiene, and dietary measures) was prescribed.

All subjects received advice and information regarding diet and nutrition at the Dietetic Department of the Diabetes Care Centre of the Institute of Endocrinology, where their diets were drawn up based on their daily calorie intake requirement per kilogram body weight and their level of physical activity. Individuals were provided with diets with the following proportion of nutrients: 55–60% carbohydrates, 15–20% protein, and 20% fat. Diets ranged from 1200 to 1500 cal [49]. Patients in both groups were also encouraged to increase their physical activity (walking for 30–45 min a day, 3–4 days a week) [49].

Several interim assessments were performed at 1.5, 3.0, and 4.5 months, including physical examination, anthropometric measurements (weight, height, waist, and hip circumference), physical activity through the IPAQ questionnaire [50], diet adherence (24-h dietary recall), and the evaluation of frequencies of adverse events. At 6 months, the same assessments as above were performed, in addition to biochemical determinations [fasting plasma glucose, lipid profile (total cholesterol, triglycerides, HDL-c and low-density lipoprotein cholesterol (LDL-c)], creatinine, uric acid, and liver enzymes, as well as blood glucose levels at two hours after a glucose tolerance test). Non-compliance with treatment (Obex® or placebo) or discontinuation criteria were considered when the subject failed to consume more than 20 consecutive or single doses of the study product between assessments.

Randomisation within the study was designed using a computerised random number generator. All personnel involved in the study remained unaware of the correspondence between the codes and the content of the sachets. The treatments used for the study (Obex® and placebo) were provided by Laboratorios Catalysis, labeled with the randomisation code only. Code-to-sachet content associations were kept in a sealed envelope under the custody of the Head of the Research Methodology Department of the Institute of Endocrinology. Seal and envelope integrity was checked every three months. At the end of the study, the envelope was opened.

Individual assessment of response was performed after three and six months of treatment. The complete success of weight and waist circumference reduction was considered to be when participants achieved a ≥ 5% reduction in both weight and waist circumference relative to the baseline value; partial success was when subjects achieved a ≥ 5% reduction in either of the two main anthropometric measurements (weight or waist circumference), and failure was considered to be when subjects did not achieve a ≥ 5% reduction in either weight or waist circumference or when an increase in both parameters was observed.

At 12 weeks, subjects in both groups who had a reduction ≥ 5% of their body weight and waist circumference were categorised as early responders and continued participating in the study for up to 24 weeks. Once the optimal early response criterion was identified, subjects were classified as early responders (ERs) or early non-responders (ENRs) [51]. Early responders refers to individuals who simultaneously achieved weight loss and a reduction of waist circumference ≥ 5% from the baseline assessment at 3 months and early non-responders refers to individuals who did not simultaneously achieve weight loss and a reduction of waist circumference ≥ 5% from the baseline assessment at 3 months.

The study was monitored by the National Coordinating Centre for Clinical Trials (Centro Nacional Coordinador de Ensayos Clínicos, CENCEC). The clinical trial began in November 2018 (first person in) and lasted until June 2019 (last person completed).

### Study product

The dietary supplement Obex® used in this study was supplied by Laboratorios Catalysis. This product is marketed as a food supplement by Laboratorios Catalysis (Macarena, no. 14 28,016 Madrid, Spain). The sachets of Obex® (4 g) contained the ingredients specified in Table 1. Obex® dosage was obtained from previous studies [46–48].

**Table 1:** Qualitative-quantitative composition of Obex®

Ingredients	mg
*Caralluma fimbriata* extract	1500
Maltodextrin	682
Inositol	500
Pineapple flavour	396.8
Choline bitartrate	200
L-methionine	200
L-Arginine	144
*Phaseolus vulgaris* L. extract	100
Inulin	100
Acai berry extract (*Euterpe oleracea Mart.*)	50
L-ornithine	50
Citric acid	50
L-carnitine fumarate	13
Zinc sulphate	7
Sucralose	3.2
Calcium pantothenate	3
Vitamin B6 (Pyridoxine hydrochloride)	0.9
Folic acid (Pteroylmonoglutamic acid)	0.1

Nutritional analysis of 100 g of Obex® showed that it contained 377 kcal (1,602 kJ) as the energy value, 77.3 g of carbohydrates, 12.6 g of proteins and 2.0 g of total fats

After the initial assessment, all subjects who met the eligibility criteria and wished to participate in the study were subsequently enrolled. Subjects were randomly assigned to receive either Obex® (80) or placebo (*n* = 80). The participants took Obex® or placebo at a dose of one sachet 15 to 20 min before the two main meals (lunch and dinner) daily (mean two sachets per day) for six months while maintaining a diet appropriate to their weight and level of physical activity, as well as appropriate hypertensive drugs (angiotensin-converting enzyme inhibitors) in the case of subjects with hypertension. Catalysis advised dissolving the contents of the sachet in a glass of water or fruit juice (sugar-free homemade fruit juice), never with dairy products.

The effects of Obex® were assessed three and six months after the start of treatment and compared with the placebo group**.**

### Adverse events

Every one and a half months, a clinical examination of the participants was performed to determine whether they experienced any adverse events. Adverse events such as rashes, headache, diarrhoea, nausea, dyspepsia, and bloating were recorded.

### Physical examination

The physical examination included assessments of height, weight, waist and hip circumference, and blood pressure. The physical assessments were performed at baseline, 3 and 6 months after starting Obex® or placebo.

Height and weight were measured, and body mass index (BMI) was calculated as weight (kg) divided by height squared (m^2^) (kg/m^2^). Overweight and obesity were defined and evaluated using BMI and World Health Organisation (WHO) cut-off points (BMI 25–29.9 kg/m^2^ for overweight and BMI 30 to < 40 kg/m^2^ for obesity).

Waist circumference (WC) was measured with the person standing, with an inelastic tape at the midpoint between the lower margin of the rib cage and the superior iliac crest, during mild expiration. Waist-to-hip ratio (WHR) was defined as the ratio of waist girth to the circumference of the hips measured at the greater trochanter. Measurements were obtained in duplicate, and their averages were used for the analysis. The waist/height ratio (WHTR) was calculated as WC (cm) divided by height (cm). The conicity index was calculated according to the formula proposed by Valdez [52].

Blood pressure was measured three times after a 5-min rest in the sitting position using standard mercury sphygmomanometers [53] by following the Proper Measurement of Clinic Blood Pressure [54]. Systolic blood pressure (SBP) and diastolic blood pressure (DBP) were measured at the first and fifth Korotkoff phases, respectively. The average of the three blood pressure measurements was recorded and included in the analysis.

### Laboratory tests

At the start of the study (baseline) and six months, venous blood samples were collected after an overnight fast for biochemical assessments: fasting plasma glucose, lipid profile (total cholesterol, triglycerides, HDL-cholesterol and LDL-cholesterol), creatinine, uric acid and hepatic enzymes, as well as a second blood sample taken 2 h after the oral glucose tolerance test (OGTT), and the values were recorded. In addition, fasting insulin concentrations were also measured.

The fasting glucose and insulin concentrations were calculated for each subject on two separate occasions: at baseline and 5 min. To calculate the insulin resistance index (HOMA-IR), insulin secretion index (HOMA-β) and insulin sensitivity indexes (IS), the averages of the fasting glucose and insulin values were obtained at baseline and after 5 min.

The insulin resistance index was calculated using the Matthews homeostasis model assessment (HOMA-IR) (fasting insulin μU/ml x fasting glucose mmol/l / 22.5) [55, 56] Insulin secretion (HOMA-β) was also calculated by assessment of the homeostatic model (20 × fasting insulin (μU/ml) / fasting glucose (mmol/l)—3.5) [55–57].

Three indirect indices were used to calculate the insulin sensitivity (IS). They were calculated according to the following formulae:

- Quantitative Insulin Sensitivity Check Index (QUICKI) = 1 / [log fasting insulin_0_ + log fasting glucose_0_] [56, 57].
- Bennett index (BEN) = 1 / (log fasting insulin_0_ x log fasting glucose_0_) [56, 57].
- Raynaud index (RAY) = [40 / fasting insulin_0_] [56, 57].

Fasting and 2-h plasma glucose and lipid profile, including total cholesterol, triglycerides, HDL-cholesterol and LDL-cholesterol, in addition to creatinine and uric acid (UA), as well as hepatic enzymes and haemoglobin, were measured enzymatically using a biochemistry analyser (Mindray BS-200E, China) using commercial kits from C.P.M. Diagnostic Research (Italy) (http://www.cpmsas.it/catalogo.php?categoria=6).

The fasting plasma insulin concentration was measured by immunoradiometric assay (IRMA, Izotop, Hungary) (http://www.izotop.hu/pdf/immuno/rk400ct_a.pdf).

### Outcomes

The primary outcome was a change in anthropometric measurements and glucose, insulin and lipid concentrations, as well as variations in insulin release, insulin resistance, and insulin sensitivity. Secondary outcomes were variation in serum creatinine, uric acid, hepatic enzymes and change in blood pressure. The change (variation) was the difference obtained between baseline and the end of treatment (6 months).

Another primary outcome was variation in the biochemical measurements, as well as differences in insulin release, insulin resistance and insulin sensitivity between the ERs and ENRs for each treatment group (Obex® and placebo), in addition to the change obtained between baseline and 6 months of treatment in the ERs and ENRs in each treatment group among subjects who completed the trial.

### Statistical analysis

Data were expressed as mean ± standard deviation and as percentages for categorical variables (before and after). Differences between groups (Obex® and placebo) were analysed by Mann–Whitney nonparametric independent sample test.

Wilcoxon signed-rank tests were used to compare changes between baseline and the end of treatment (6 months) and the Friedman test was used for several related samples. In addition, both Pearson's Chi-square test and Fisher's exact test were used for categorical variables.

Mann–Whitney tests were also used to compare baseline values between ERs and ENRs within each treatment group (Obex® and placebo). Wilcoxon-rank sum tests were utilised to compare changes at baseline and after six months of treatment between ERs and ENRs within each group.

All significance tests and resulting *p* values were two-sided, with an alpha level of 0.05. Statistical analyses were performed using SPSS version 19 (SPSS Inc., Chicago, IL, USA).

## Results

**Fig. 1:**
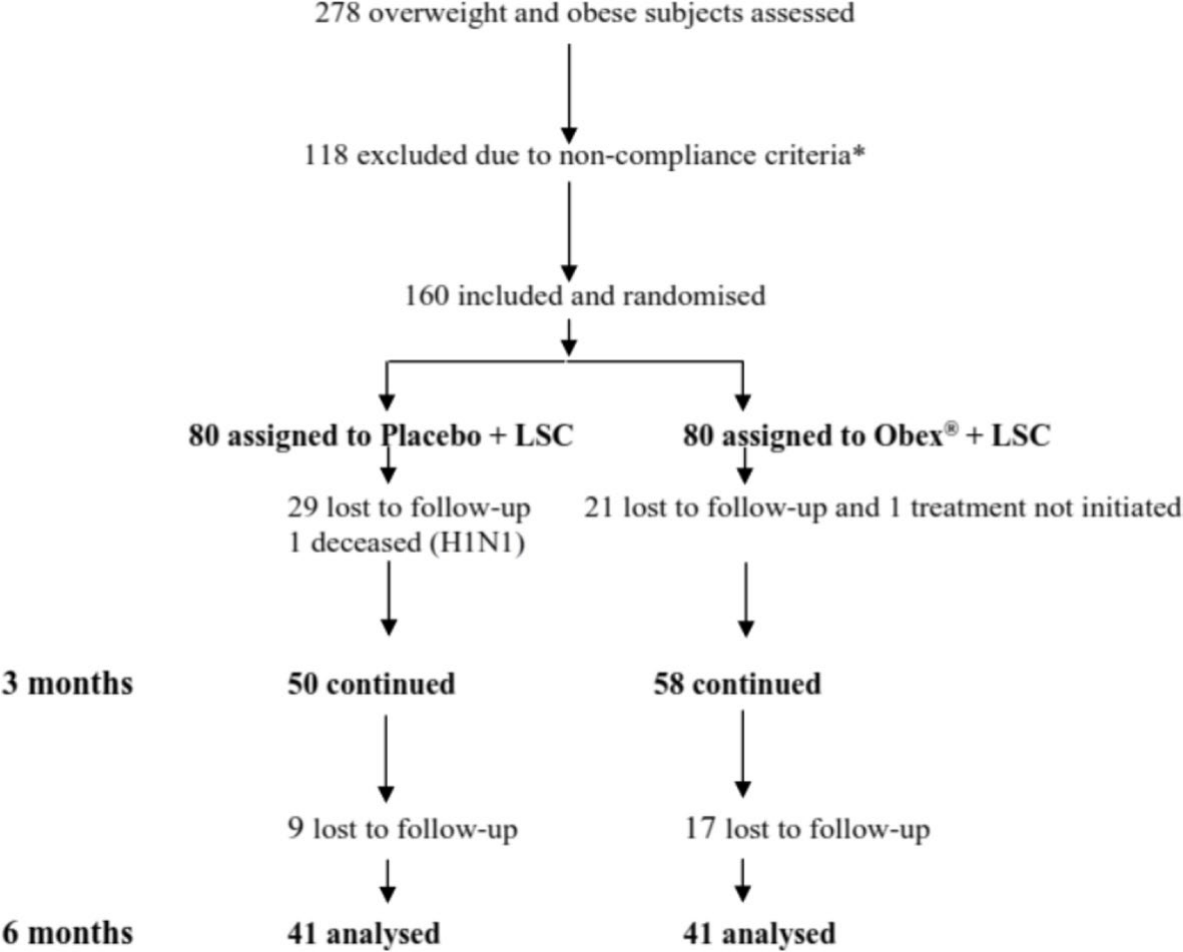
Flow chart showing subject distribution throughout the study. LSC: lifestyle change. *Excluded due to: Gynoid obesity (35), morbid obesity (30), lack of laboratory analysis (23), anaemia (6), hypothyroidism (6), unknown DM2 (5), subjects in treatment (5), PCOS (3), elevated liver enzymes (3), age (2)

Of the 278 overweight and obese subjects screened between 2018 and 2019 for the study at the Endocrinology Institute, 160 met the criteria to enter the study and underwent randomisation, as indicated in Fig. 1. For this analysis, 160 subjects were randomly assigned to two groups of an equal number, one group (80) receiving Obex® and the other group (80) receiving placebo, plus non-pharmacological treatment [physical activity (PA) and nutritional counselling (NC)] for six months. The distribution of the trial participants at baseline, at 3 months and 6 months is shown in Fig. 1.

Among the participants, 51.3% (82/160) completed the trial up to 6 months. During the trial, 48.7% (78/160) were lost to follow-up (non-initiation or non-compliance with treatment, prolonged visit abroad and viral infections) (Fig. 1). Of the total number of subjects who did not complete the study in each treatment group (Obex® or placebo), 18.4% (7/38) were non-compliant (14 subjects discontinued for missing more than 20 consecutive doses during the trial period).

The distribution of age, gender, skin colour and the presence of acanthosis nigricans was similar in both groups. There were no significant differences between the placebo and Obex® groups for any clinical and biochemical variables at baseline, except for age, which was higher in the Obex® group (Table 2).

**Table 2:** Baseline characteristics of the participants

Characteristics	Obex® (*n* = 80)	Placebo (*n* = 80)
Women [n (%)]	70 (87.5)	69 (86.3)
Skin colour (White) [n (%)]	55 (68.8)	56 (70.0)
Obese [n (%)]	62 (77.5)	67 (83.8)
Acanthosis nigricans [n (%)]	38 (47.5)	40 (50.0)
	**Mean (SD)**	**Mean (SD)**
Age (years)	45.1 (11.9)*	40.8 (10.8)
Weight (kg)	86.1 (11.9)	86.4 (12.4)
Height (cm)	161.5 (7.2)	160.8 (7.4)
BMI (kg/m^2^)	32.9 (3.4)	33.3 (3.3)
Waist circumference (cm)	105.2 (8.8)	104.8 (9.6)
Hip circumference (cm)	113.3 (7.5)	113.1 (8.0)
Waist-hip ratio	0.93 (0.05)	0.93 (0.05)
Waist-height ratio	0.65 (0.05)	0.65 (0.06)
Conicity index	1.32 (0.07)	1.31 (0.07)
Systolic blood pressure (mmHg)	114.8 (13.6)	117.1 (15.8)
Diastolic blood pressure (mmHg)	72.2 (11.6)	73.7 (8.8)
Haemoglobin (g/l)	129.2 (11.4)	127.2 (8.8)
Fasting blood glucose (mmol/l)	5.4 (0.52)	5.3 (0.53)
Blood glucose 2 h after OGTT (mmol/l)	6.0 (1.6)	5.9 (1.4)
HbA1c (%)	5.2 (0.68)	5.3 (0.70)
Fasting insulin (µU/ml)	17.0 (10.1)	17.1 (9.0)
HOMA-IR	4.2 (2.8)	4.1 (2.5)
HOMA-β	185.5 (91.2)	191.4 (95.8)
QUICKI	0.54 (0.07)	0.54 (0.07)
Bennett	1.23 (0.31)	1.23 (0.32)
Raynaud	0.43 (0.26)	0.43 (0.25)
Total cholesterol (mmol/l)	5.5 (1.2)	5.3 (1.1)
Triglycerides (mmol/L)	1.9 (1.0)	1.8 (0.9)
HDL- cholesterol (mmol/l)	1.57 (0.39)	1.51 (0.35)
LDL- cholesterol (mmol/l)	3.38 (1.1)	3.23 (0.9)
Creatinine (µmol/l)	86.1 (12.7)	84.3 (14.4)
Uric acid (µmol/l)	307.6 (79.7)	307.6 (78.1)
ALT (IU/l)	28.6 (13.9)	27.7 (12.9)
AST (IU/l)	25.2 (9.5)	23.4 (8.0)
GGT (IU/l)	30.8 (26.1)	30.4 (25.1)

*SD* Standard deviation, *BMI* body mass index, *HDL-c* high-density lipoprotein cholesterol, *LDL-c* low-density lipoprotein cholesterol, *HOMA-IR*, homeostasis model assessment of insulin resistance, *HOMA-β* homeostasis model assessment for β-cell function, *QUICKI* quantitative insulin sensitivity check index, *BENNETT* and *RAYNAUD* insulin sensitivity indexes, *ALT* Alanine transaminase, *AST* Aspartate transaminase, *GGT* Gamma-glutamyltransferase, *OGTT* Oral Glucose Tolerance Test

^*^p = 0.007 vs placebo

Both the Obex® and placebo groups achieved a reduction in all the anthropometric measurements (weight, BMI, waist and hip circumference, waist-to-hip ratio, waist-to-height ratio and conicity index) over the 6 months of the study (Table 3). However, blood pressure was not decreased compared to baseline values during this period in either of the groups (Table 3).

**Table 3:** Anthropometric and blood pressure changes in overweight and obese people during the follow-up period

Characteristics	**Obex®**			**Placebo**		
	**Baseline** (*n* = 80) Mean (SD)	**After 3 m** (*n* = 58) Mean (SD)	**After 6 m** (*n* = 41) Mean (SD)	**Baseline** (*n* = 80) Mean (SD)	**After 3 m** (*n* = 50) Mean (SD)	**After 6 m** (*n* = 41) Mean (SD)
Weight (kg)	86.1 (11.9)	80.8 (10.3)	**79.5 (11.1)**^**a**^	86.4 (12.4)	80.8 (11.9)	**78.6 (12.0)**^**a**^
BMI (kg/m^2^)	32.9 (3.4)	31.2 (3.3)	**30.6 (3.7)**^**a**^	33.3 (3.3)	31.2 (3.4)	**30.3 (3.3)**^**a**^
Waist circumference (cm)	105.2 (8.8)	98.1 (8.0)	**96.4 (8.8)**^**a**^	104.8 (9.6)	98.1 (8.8)	**95.1 (9.1)**^**a**^
Hip circumference (cm)	113.3 (7.5)	111.6 (6.9)	**110.3 (7.6)**^**a**^	113.1 (8.0)	111.9 (7.5)	**110.5 (7.6)**^**b**^
Waist-hip ratio	0.93 (0.05)	0.88 (0.07)	**0.87 (0.07)**^**a**^	0.93 (0.05)	0.88 (0.06)	**0.86 (0.06)**^**a**^
Waist-height ratio	0.65 (0.05)	0.61 (0.05)	**0.60 (0.06)**^**a**^	0.65 (0.06)	0.61 (0.05)	**0.59 (0.05)**^**a**^
Conicity index	1.32 (0.07)	1.27 (0.07)	**1.26 (0.08)**^**a**^	1.31 (0.07)	1.27 (0.07)	**1.25 (0.07)**^**a**^
Systolic BP (mmHg)	114.8 (13.6)	116.7 (11.3)	119.4 (10.4)	117.1 (15.8)	115.7 (13.9)	119.3 (9.5)
Diastolic BP (mmHg)	72.2 (11.6)	70.8 (9.2)	70.7 (7.9)	73.7 (8.8)	69.5 (7.9)	70.4 (7.2)

*M* months, *SD* Standard deviation, *BMI* Body mass index, *BP* Blood pressure

^a^ *p* < 0.0001; ^b^ *p* = 0.005 Friedman test

After 6 months of treatment, no significant changes in most biochemical measurements were observed between the groups, except for HDL-c levels, which were higher in subjects receiving Obex® compared to placebo (*p* = 0.030). Furthermore, we found a reduction of glucose levels at 2 h during the OGTT (*p* = 0.035) and AST levels (*p* = 0.012) in the placebo group compared to Obex® (Table 4). Over the same time period, both groups achieved a reduction in cholesterol and triglyceride concentrations with respect to the baseline value (Table 4). However, those intake Obex® had reduced fasting insulin concentrations at 6 months below baseline values [16.9 ± 8.9 μU/ml to 14.3 ± 7.9 μU/ml (*p* = 0.035)] (Table 4). Likewise, a decrease in creatinine (*p* = 0.001) and uric acid (*p* = 0.004) concentrations with respect to the initial value was observed in the Obex®-treated subjects, which was not the case in the placebo group. Additionally, a decrease in ALT levels appeared in the placebo group at 6 months with respect to the initial values (*p* < 0.0001) (Table 4).

**Table 4:** Evolution of biochemical measurements in overweight and obese individuals during the follow-up period

Measurements	Obex® *n* = 41			Placebo *n* = 41	
	**Baseline** Mean (SD)	**After 6 m** Mean (SD)		**Baseline** Mean (SD)	**After 6 m** Mean (SD)
Haemoglobin (g/l)	130.9 (10.3)	128.7 (11.0)		127.9 (9.0)	128.7 (11.7)
Fasting blood glucose (mmol/l)	5.4 (0.6)	5.3 (0.5)		5.3 (0.5)	5.3 (0.4)
Glucose at 2 h OGTT (mmol/l)	6.2 (1.6)	6.5 (1.5)		5.9 (1.2)	**5.9** (1.2)^i^
Fasting insulin (µU/ml)	16.9 (8.9)	**14.3** (7.9)^a^		15.2 (8.0)	13.2 (5.1)
HOMA-IR	4.1 (2.4)	**3.4** (1.8)^b^		3.6 (1.9)	3.1 (1.3)
HOMA-β	187.8 (91.3)	168.4 (107.2)		177.3 (105.0)	152.2 (53.9)
QUICKI	0.54 (0.08)	0.55 (0.07)		0.55 (0.08)	0.56 (0.05)
Bennett	1.25 (0.39)	1.31 (0.33)		1.29 (0.36)	1.30 (0.22)
Raynaud	0.44 (0.32)	**0.48** (0.26)^c^		0.48 (0.29)	0.48 (0.17)
Total cholesterol (mmol/l)	5.7 (1.1)	**5.3** (0.9)^d^		5.5 (1.0)	**5.0** (0.9)^j^
Triglycerides (mmol/L)	1.8 (0.9)	**1.6** (1.1)^e^		1.8 (0.9)	**1.4** (0.7)^**j**^
HDL- cholesterol (mmol/l)	1.64 (0.4)	**1.74** (0.3)^h^		1.62 (0.4)	1.58 (0.3)
LDL- cholesterol (mmol/l)	3.7 (1.1)	3.7 (0.9)		3.4 (0.8)	3.4 (0.8)
Creatinine (µmol/l)	87.0 (14.0)	**81.0** (12.7)^f^		84.3 (18.0)	80.6 (14.2)
Uric acid (µmol/l)	309.9 (84.9)	**285.9** (77.7)^g^		293.6 (83.7)	294.2 (96.0)
ALT (IU/l)	29.1 (13.4)	28.2 (22.1)		28.3 (15.6)	**21.6** (12.1)^j^
AST (IU/l)	25.3 (7.9)	28.8 (14.2)		24.0 (9.0)	**22.5** (6.0)^k^
GGT (IU/l)	35.4 (31.1)	36.2 (29.6)		32.7 (31.3)	33.4 (30.1)

*M* months, *SD* Standard deviation, *h* hours. Data are given as mean (SD), *SD* standard deviation; *n:* total cases studied in each of the groups during the follow-up period analysed, *HDL-c* high-density lipoprotein cholesterol, *LDL-c* low-density lipoprotein cholesterol, *HOMA-IR* homeostasis model assessment of insulin resistance, *HOMA-β* homeostasis model assessment for β-cell function, *QUICKI* quantitative insulin sensitivity check index, *BENNETT* and *RAYNAUD* insulin sensitivity indexes, *ALT* Alanine transaminase, *AST* Aspartate transaminase, *GGT* Gamma-glutamyltransferase, *OGTT* Oral Glucose Tolerance Test

^a^ *p* = 0.035

^b^*p* = 0.049

^c^ *p* = 0.036

^d^ *p* = 0.008

^e^*p* = 0.011

^f^ *p* = 0.001

^g^ *p* = 0.004

^j^*p* < 0.0001 vs Baseline, Wilcoxon signed-rank test

^h^ *p* = 0.030 vs Placebo 6 months, Mann–Whitney test

^i^*p* = 0.035

^k^*p* = 0.012 vs Obex 6 months, Mann-Whitney test

Table 4 also shows several indexes related to insulin sensitivity and insulin secretion calculated at baseline and after receiving Obex® or placebo for 6 months. After 6 months of treatment, only subjects in the Obex® group exhibited a significant change with respect to baseline in HOMA-IR values (*p* = 0.049) and Raynaud's index (*p* = 0.036). However, no differences were found between groups after 6 months of treatment regarding HOMA-IR, HOMA-β and the three indexes of insulin sensitivity (Table 4).

Both treatments (Obex® and placebo) helped to reduce anthropometric measurements through the action of physical activity and diet. Also, they decreased cholesterol and triglyceride concentrations compared to baseline values after 6 months of therapy (Tables 3 and 4). The proportion of participants in the Obex® and placebo groups who met the moderate/high physical activity goal as assessed by the IPAQ questionnaire at 3 months was similar [77.6% (45/58) and 82.0% (41/50), respectively, versus 73.2% (30/41) and 68.3% (28/41) at 6 months]. There was no significant difference between the Obex® and placebo groups in terms of dietary compliance at 3 months and at the end of the study [65.5% (38/58) and 64.0% (32/50) at 3 months, and 70.7% (29/41) and 75.6% (31/41) at 6 months, respectively].

After receiving Obex® for 3 months, 48.3% (28/58) of the participants achieved complete success (reduction ≥ 5% in both weight and waist circumference relative to the baseline value), as opposed to individuals receiving placebo who revealed a lower complete success rate [26.0% (13/50), *p* = 0.022]. However, at six months there was no difference between the two groups in terms of treatment success (Table 5).

**Table 5:** Individual assessment of weight loss and reduction of waist circumference in both treatment groups during the follow-up period

Individual assessment	Obex®		Placebo	
	**3 months** (*N* = 58) n (%)	**6 months** (*N* = 41) n (%)	**3 months** (*N* = 50) n (%)	**6 months** (*N* = 41) n (%)
Complete success	**28 (48.3)**^**a**^	27 (65.9)	13 (26.0)	26 (63.4)
Partial success	**16 (27.6)**	7 (17.1)	26 (52.0)	8 (19.5)
Failure	**14 (24.1)**	7 (17.1)	11 (22.0)	7 (17.1)

Complete success: individuals with a reduction ≥ 5% in both weight and waist circumference from baseline assessment

Partial success: individuals with a reduction ≥ 5% in either of the two main anthropometric measures (weight or waist circumference)

Failure: individuals with no reduction in weight or waist circumference, or an increase in both parameters

^a^ *p* = 0.022 vs Placebo 3 months Pearson's Chi-square test

Among individuals receiving Obex® who reached the end of the trial, 53.7% (22/41) were ERs and 46.3% (19/41) were ENRs. The proportion of individuals who were ERs in the placebo group was much lower than Obex® treated subjects [31.7% (13/41)] (Table 6). The baseline characteristics of all ERs and ENRs for each group recruited in this study are described in Table 6.

**Table 6:** Baseline characteristics of participants with early responder or early non-responder status in each study group

Characteristics	**Obex®**			**Placebo**		
	**All** (*n* = 41)	**ERs** (*n* = 22)	**ENRs** (*n* = 19)	**All** (*n* = 41)	**ERs** (*n* = 13)	**ENRs** (*n* = 28)
Women [n (%)]	52 (85.4)	21 (95.5)	14 (73.7)	34 (82.9)	12 (92.3)	22 (78.6)
Skin colour (White) [n(%)]	28 (68.3)	14 (63.6)	14 (73.7)	30 (73.2)	8 (61.5)	22 (78.6)
Obese [n (%)]	32 (78.0)	19 (86.4)	13 (68.4)	34 (82.9)	11 (84.6)	23 (82.1)
Acanthosis nigricans [n(%)]	23 (56.1)	13 (59.1)	10 (52.6)	19 (46.3)	8 (61.5)	11 (39.3)
	**Mean (SD)**	**Mean (SD)**	**Mean (SD)**	**Mean (SD)**	**Mean (SD)**	**Mean (SD)**
Age (years)	49.0 (10.6)	47.6 (10.7)	**50.5 (10.5)**^**a**^	44.7 (9.2)	46.4 (8.6)	43.9 (9.5)
Weight (kg)	87.1 (10.5)	86.3 (9.6)	88.0 (11.6)	85.4 (13.4)	84.7 (13.0)	85.7 (13.8)
BMI (kg/m^2^)	33.6 (3.5)	33.6 (3.1)	33.5 (3.9)	33.0 (3.5)	33.2 (3.5)	32.9 (3.6)
Waist circumference (cm)	106.5 (7.4)	106.0 (5.8)	107.2 (9.1)	103.9 (8.8)	105.2 (10.4)	103.3 (8.1)
Hip circumference (cm)	114.4 (7.3)	115.2 (7.1)	113.4 (7.6)	112.5 (7.6)	114.7 (10.1)	111.5 (6.0)
Waist-hip ratio	0.93 (0.05)	0.92 (0.06)	0.95 (0.05)	0.92 (0.04)	0.92 (0.03)	0.93 (0.05)
Waist-height ratio	0.66 (0.04)	0.66 (0.04)	0.66 (0.05)	0.65 (0.06)	0.66 (0.06)	0.64 (0.06)
Conicity index	1.33 (0.07)	1.33 (0.07)	1.34 (0.08)	1.31 (0.07)	1.33 (0.06)	1.30 (0.07)
Systolic BP (mmHg)	117.9 (13.9)	117.0 (14.7)	119.1 (13.2)	118.9 (18.5)	124.6 (29.3)	116.2 (10.0)
Diastolic BP (mmHg)	75.3 (11.2)	73.1 (11.9)	77.9 (10.2)	74.8 (8.8)	**78.9 (9.4)**^**b**^	72.9 (8.0)

*SD* Standard deviation, *BMI* Body mass index, *BP* Blood pressure, *ERs* Early responders (individuals who simultaneously achieved weight loss and reduction of waist circumference ≥ 5% from baseline assessment at 3 months)

ENRs: Early non-responders (individuals who did not simultaneously achieve weight loss and/or reduction of waist circumference ≥ 5% from baseline assessment at 3 months)

^a^*p* = 0.006 vs Placebo ENRs; ^b^*p* = 0.033 vs Placebo ENRs Mann–Whitney test

Particularly, large improvements were observed at 6 months in Obex® ERs versus ENRs. During this period, when comparing Obex® ERs to Obex® ENRs, we found that ERs had a significant reduction in insulin, triglycerides, HOMA-IR, and HOMA-β values in addition to an increase in the three insulin sensitivity indexes compared to ENRs (Table 7).

**Table 7:** Changes in the mean values of biochemical measurements according to early responder or early non-responder status in each study group

**Characteristics**	**Obex®**				**Placebo**			
	**ERs** (*n* = 22)		**ENRs** (*n* = 19)		**ERs** (*n* = 13)		**ENRs** (*n* = 28)	
	Baseline	6 months	Baseline	6 months	Baseline	6 months	Baseline	6 months
Haemoglobin (g/l)	131.6 (10.6)	128.4 (11.8)	129.7 (9.9)	129.1 (10.3)	128.5 (8.3)	126.8 (11.5)	127.6 (9.5)	128.5 (12.8)
Fasting blood glucose	5.4 (0.6)	5.2 (0.5)	5.4 (0.5)	5.43 (0.5)	5.4 (0.6)	5.3 (0.5)	5.3 (0.4)	5.3 (0.3)
Glucose at 2 h OGTT (mmol/l)	6.3 (1.8)	6.5 (1.0)	6.1 (1.4)	6.7 (2.0)	6.0 (1.6)	5.9 (1.3)	5.8 (1.0)	5.9 (1.2)
Fasting insulin (µU/ml)	15.1 (7.2)	**11.5 (3.9)**^**a#**^	18.7 (10.2)	17.4 (9.9)	15.1 (6.5)	12.6 (5.3)	15.3 (8.7)	13.5 (5.1)
HOMA-IR	3.6 (1.8)	**2.7 (1.0)**^**b&**^	4.6 (2.8)	4.1 (2.2)	3.7 (1.7)	3.0 (1.5)	3.6 (2.0)	3.2 (1.3)
HOMA-β	170.5 (85.9)	**135.8 (53.1)**^c^	202.2 (97.7)	204.5 (138.4)	163.7 (72.3)	141.8 (45.7)	183.7 (117.9)	157.1 (57.5)
QUICKI	0.55 (0.08)	**0.58 (0.07)**^**b¥**^	0.53 (0.07)	0.53 (0.05)	0.55 (0.10)	0.56 (0.05)	0.55 (0.07)	0.55 (0.05)
Bennett	1.30 (0.44)	**1.41 (0.38)**^**b£**^	1.19 (0.31)	1.19 (0.21)	1.32 (0.52)	1.33 (0.23)	1.28 (0.26)	1.29 (0.21)
Raynaud	0.48 (0.37)	**0.56 (0.30)**^**aβ**^	0.40 (0.25)	0.39 (0.16)	0.49 (0.40)	0.50 (0.17)	0.47 (0.23)	0.47 (0.18)
Total cholesterol (mmol/l)	5.6 (1.3)	5.4 (1.1)	5.8 (1.0)	**5.2 (0.7)**^**∞**^	5.5 (0.9)	**5.0 (1.1)**^**¶**^	5.5 (1.1)	**5.0 (0.8)**^**†**^
Triglycerides (mmol/L)	1.7 (1.0)	**1.3 (0.6)**^**d€**^	2.0 (0.7)	2.0 (1.4)	1.6 (0.5)	**1.0 (0.4)**^**e§**^	1.9 (1.0)	**1.5 (0.7)**^**†**^
HDL- cholesterol (mmol/l)	1.61 (0.42)	1.74 (0.38)	1.64 (0.48)	1.75 (0.32)	1.69 (0.45)	1.65 (0.32)	1.58 (0.34)	1.56 (0.29)
LDL- cholesterol (mmol/l)	3.59 (1.15)	3.77 (0.94)	3.73 (1.01)	3.58 (0.91)	3.27 (0.54)	3.47 (0.99)	3.45 (0.88)	3.43 (0.69)
Creatinine (µmol/l)	84.4 (12.5)	81.6 (10.3)	89.1 (15.7)	**80.3 (15.3)**^**∆**^	84.9 (16.7)	78.1 (9.5)	84.1 (18.8)	81.8 (16.0)
Uric acid (µmol/l)	301.6 (88.9)	275.0 (78.1)	327.4 (84.9)	**297.8 (77.5)**^**∑**^	271.4 (53.3)	259.5 (62.5)	303.9 (93.6)	310.3 (105.2)
ALT (IU/l)	25.9 (8.9)	27.8 (26.2)	32.0 (16.9)	28.6 (17.3)	28.0 (14.6)	22.5 (13.9)	28.4 (16.4)	**21.2 (11.4)**^**‡**^
AST (IU/l)	24.0 (6.1)	27.7 (12.2)	26.3 (9.8)	30.1 (16.3)	24.3 (10.5)	23.5 (5.3)	23.8 (8.4)	22.0 (6.3)
GGT (IU/l)	27.9 (26.9)	**34.1 (33.1)**^**&**^	43.0 (33.7)	38.5 (25.8)	37.5 (44.0)	42.5 (49.1)	30.5 (24.1)	29.1 (14.6)

*ERs* Early responders, *ENRs* Early non-responders; Data are given as mean (SD), *SD* standard deviation, *HDL-c* high-density lipoprotein cholesterol, *LDL-c* low-density lipoprotein cholesterol, *OGTT* Oral Glucose Tolerance Test, *HOMA-IR* homeostasis model assessment of insulin resistance, *HOMA-β* homeostasis model assessment for β-cell function; QUICKI: quantitative insulin sensitivity check index, BENNETT and RAYNAUD: insulin sensitivity indexes; ALT: Alanine transaminase; AST: Aspartate transaminase; GGT: Gamma-glutamyl transferase

^a^*p* = 0.014; ^b^*p* = 0.011; ^c^*p* = 0.026; ^d^*p* = 0.023; ^e^*p* = 0.008 vs ENRs at 6 months, Mann–Whitney test

^†^*p* = 0.001; ^‡^*p* < 0.0001 vs Placebo ENRs baseline; ^¶^*p *= 0,019; ^§^*p* = 0.003 vs Placebo ERs baseline, Wilcoxon signed-rank test

^#^*p* = 0.016; ^&^*p* = 0.015; ^¥^ *p* = 0.042, ^£^*p* = 0.046; ^β^*p* = 0.025; ^€^*p* = 0.005 vs Obex ERs baseline; ^∞^*p* = 0.018; ^∆^ *p* = 0.002; ^∑^*p* = 0.005 vs Obex ENRs baseline, Wilcoxon signed-rank test

Additionally, Obex® ENRs showed a significant reduction in total cholesterol (*p* = 0.018), creatinine (*p* = 0.002) and uric acid (*p* = 0.005) concentrations at 6 months versus baseline (Table 7). After 6 months, ERs treated with Obex® exhibited a rise in GGT levels with respect to baseline values (*p* = 0.015) (Table 7).

Most changes in biochemical variables were not favourable in placebo-treated ERs or ENRs, except for triglyceride and cholesterol concentrations, which decreased in both ERs and ENRs relative to baseline in each group (Table 7). Nevertheless, triglyceride concentrations in the ERs with placebo diminished much more than in the ENRs (*p* = 0.008) (Table 7). Over the same period, ENRs with placebo reduced alanine aminotransferase (ALT) levels relative to baseline (*p* < 0.0001) (Table 7).

Obex® was safely administered. No serious or mild adverse events such as rash, headache, dyspepsia and bloating were observed in any participant during the trial. In the Obex® group, nausea was reported in only one case. In contrast, in the placebo group, there was one non-product-related serious adverse event, and three other adverse events such as epigastric pain, bloating and vomiting.

## Discussion

Weight loss can ameliorate or even reverse significant metabolic sequelae in overweight individuals [58]. Understanding the heterogeneity of weight loss response to specific drugs, as well as to diet and physical activity, has tremendously significant clinical and public health implications [51]. The present study examines the patterns of weight loss associated with treatment with Obex® and placebo twice daily, plus non-pharmacological treatment consisting of lifestyle changes (physical activity and hygiene and dietary measures) in overweight and obese people without diabetes.

One of the most worrying aspects related to obesity is the high treatment dropout rate [59]. The dropout rate per year in most weight loss studies ranges from 40 to 80% [51, 60, 61]. The proportion of dropouts at 6 months in this study (48.7%) is within the range of most studies. Therefore, it seems advisable to integrate this weight loss treatment within a multidisciplinary programme of continuous care, involving professionals from the fields of dietetics and nutrition, physical activity, psychology and motivation, among others [59], in order to try to minimise the effect of abandonment in interventions for people with excess weight.

In this randomised double-blind study, we observed a similar decrease in all the anthropometric variables analysed at 3 and 6 months of treatment in both study groups (Obex® and placebo). This is the first long-term intervention of Obex® treatment compared to a placebo group in overweight and obese individuals. Previous pilot studies using Obex® demonstrated a reduction in abdominal obesity and other anthropometric measurements in individuals consuming this nutritional supplement [46, 48]. However, in this clinical trial Obex® intake was found to aid more rapid weight loss and waist circumference reduction (higher proportion of subjects with complete success at 3 months) compared to placebo plus lifestyle changes.

Research in individuals without diabetes demonstrates that exenatide, liraglutide, and semaglutide are associated with significant weight loss and improved metabolic parameters [62–64]. Recently, the high early response to long-term weight loss with exenatide was found to be similar to that achieved with a hypocaloric diet (34.8 g carbohydrate, 15.8 g protein, and 6.3 g fat) [51].

Several large-scale studies in different populations have highlighted the benefit of a lifestyle modification programme that includes weight-loss diets and moderate-intensity exercise, both in the treatment of some cardio-metabolic risk factors and in decreasing the risk of diabetes progression [21–26, 65]. Our study confirms these data, as we found that lifestyle changes during the clinical trial in both groups contribute greatly to the reduction of the different anthropometric measurements, as well as to the improvement of some of the cardio-metabolic risk factors (cholesterol and triglycerides relative to baseline values).

The improvement in HDL-c levels after 6 months of receiving Obex® is possibly due to the effect of the ingredients of the supplement [66, 67], since in a previous study we found that after three months of treatment with Obex® we obtained similar results [48]. The increase in HDL cholesterol is considered a step towards the prevention of cardiovascular diseases.

A possible explanation for the presence of higher blood glucose values at 2 h after OGTT and AST enzyme values in the Obex® group compared to placebo at the end of the trial could be the older age of the subjects who consumed Obex® (see baseline characteristics). The proportion of subjects at 6 months with impaired glucose tolerance (IGT) and altered AST values in each study group was not significant [IGT (14.6%; 6/41) and AST (7.3%; 3/41) in the Obex® group and IGT (7.3%; 3/41) and AST (0.0%; 0/41) in the placebo group].

For several years, placebo has been defined by its inert content and its use as a control in clinical trials and treatments in clinical practice. Recent research shows that placebo effects can be defined as psychological and/or physiological responses following the administration of active and non-active substances when combined with the assertion of treatment effects and that these effects can be robust in both laboratory and clinical settings [68, 69]. The authors suggested that placebo interventions can improve physical disease processes of peripheral organs more easily and effectively than biochemical processes [70].

People treated with placebo also had to change their lifestyle. The decrease in all the anthropometric measurements at 6 months of treatment, compared to the initial values, may be due to the change in their lifestyle [65] and to the psychological and/or physiological effects of the placebo itself [68–70]. This interpretation is supported by the fact that most people in the placebo group improved in the aforementioned parameters, presumably because they complied with the physical exercise and diet requirements. In fact, at the end of the clinical trial, when the type of treatment of each participant was revealed, they were surprised that they had not been given Obex®.

Treatment with Obex® in overweight and obese subjects, together with lifestyle changes, decreased insulin, uric acid, creatinine and insulin resistance (HOMA-IR) values at the end of the trial relative to baseline values while improving insulin sensitivity (Raynaud's index), which did not occur in the placebo group. This implies, therefore, that the activity of this nutritional supplement is to improve insulin homeostasis [28–49, 66, 71]. Similar results were found in a previous pilot study in overweight and obese people treated with Obex® [49]. These results confirm that reducing body weight increases insulin sensitivity [23], and they also demonstrate an additional favourable effect of Obex®.

Various studies have identified an association between hyperuricaemia and a moderate increase in glucose concentrations, insulin resistance, hyperinsulinaemia, creatinine, early kidney damage, obesity, T2D and CVD [72–75]. These findings are very interesting because treatment with Obex® at 6 months improves at the same time insulin, creatinine and uric acid concentrations, as well as insulin resistance and insulin sensitivity. This indicates that, by improving insulin concentration and enhancing insulin sensitivity with Obex®, we are helping to decrease hyperuricaemia, which affects creatinine concentration, thereby helping to prevent early kidney damage. As we reduce insulin resistance and hyperuricaemia, we also delay the onset of T2D and CVD [8, 72–75].

Gut microbiota modification profile is a potential mechanism for the effects of inulin, present in Obex®, in response to the combination with other types of prebiotics (Fructo-oligosaccharides). In summary, further studies are needed to demonstrate the exact molecular mechanisms through which Obex® have an effect on adipogenesis, insulin sensitivity, inflammation, as well as some metabolic alterations and modulation of the gut microbiota [30, 47, 67, 71, 76, 77].

A rapid reduction of weight and waist circumference was the only beneficial effect of Obex® at three months. This could be explained by the peculiarities of the Obex® ingredients on improving the lipid profile (Acai berry, carnitine, zinc, faseolamin, inulin), prevent inflammation in white adipose tissue (Acai berry), improved gut microbiota health (inulin), appetite reduction (inulin, faseolamin), weight loss (inulin, faseolamin) and increasing insulin sensitivity and β-cell function (L-arginine, carnitine, zinc, inulin) [28–46, 67, 71, 76, 77].

Carreira et al. [47] suggested that Obex® supplement decreases body weight gain by decreasing adipocyte gain in mice. These authors found that Obex® inhibited proliferation of pre-adipocytes, blocked the differentiation of pre-adipocytes into new adipocytes, and decreased the lipid load in mature adipocytes.

For all the above reasons, in the first three months of taking Obex®, a reduction of ≥ 5% in both weight and waist circumference compared to baseline is achieved more quickly, as the weight loss is mainly due to a reduction in waist circumference by decreasing adipocyte gain.

It is well documented that early response to a weight loss intervention can predict long-term weight loss [58]; this is the basis for the discontinuation guidelines for all recently approved weight loss medications [61]. Response to weight loss after 1 to 4 months has been used to predict weight loss at 1 year [58]. In this trial, at 3 months a greater proportion of subjects were early responders to weight loss and reduction of abdominal obesity (ERs) in those receiving Obex® (48.3%) than in those who used placebo (26.0%). ERs receiving Obex® achieved a decrease in triglyceride, insulin and insulin resistance (HOMA-IR) levels at the end of the trial, as well as an improvement in insulin sensitivity and β-cell function (HOMA-β) with respect to baseline values and Obex® early non-responders (ENRs), which did not occur for ERs in the placebo group, with the exception of triglycerides and cholesterol. Consequently, it is confirmed that the action of Obex® in ERs who consumed Obex® is involved in the improvement of insulin homeostasis [28–45, 66, 71].

The ERs identify those more motivated and engaged individuals [51]. Patients who have baseline factors predictive of long-term weight loss failure may benefit from additional support during the intervention. In addition, if a patient does not achieve early weight loss, further support or a transition to an alternative intervention where they may have more success may be considered [51].

The creatinine and uricaemia values in the group of ENRs treated with Obex® were seen to decrease after 6 months of treatment with respect to baseline. This implies that the effectiveness of the Obex® food supplement, due to its antioxidants properties [28–45], improves creatinine and uric acid levels in this group of people. These effects on creatinine and uricaemia were not observed in ENRs treated with placebo.

At 6 months in the placebo group, in both ERs and ENRs, there was an improvement in some lipid profile parameters (triglycerides and cholesterol) relative to baseline values, which are probably the expected effects of weight loss and reduction of abdominal obesity due to lifestyle changes [21, 58, 65]. At the same time, in ER**s** in the placebo group, the reduction in triglyceride concentrations was even greater than in ENRs.

Several articles have described the beneficial effect of various components of Obex® supplementation on improving the lipid profile (Acai berry, carnitine, zinc, inulin) and increasing insulin sensitivity and β-cell function (L-arginine, carnitine, zinc, inulin) [28–45, 66, 67, 71]. Some adverse events (AEs) have been reported with the use of conventional drugs (e.g. metformin, exenatide, liraglutide, thiazolidinediones, acarbose, orlistat, lorcaserin and phentermine/topiramate) for the treatment of obesity and prevention of type 2 diabetes in populations at high risk of diabetes [22–24, 26, 27, 51, 62–64]. It is noteworthy that almost no adverse events were detected during the treatment period, confirming the safety of Obex® for use in human trials [47, 49]. We, therefore, recommend Obex® as an adjunct therapy in obese individuals and, consequently, to prevent and/or delay the onset of type 2 diabetes. Although these drugs and Obex® are effective, they are not sufficient by themselves and the treatment must be combined with lifestyle changes. It is encouraging that no AEs occurred in ERs with Obex® and placebo compared to early non-responders.

A possible limitation of the present clinical trial is the relatively small sample size at the end of the study as a consequence of the high dropout rate. With respect to non-completers, other studies have shown that people with poor early weight loss progress tend to drop out of such weight loss programmes [78, 79].

In conclusion, daily supplementation with Obex® improved levels of selected markers of metabolic disease risk and increased cardiovascular protective effect, as well as insulin homeostasis, and was well tolerated and safe in overweight/obese adults. The results of this research highlight the importance of improved insulin sensitivity and β-cell function in early responders (ERs) treated with Obex® and lifestyle changes. Finally, long-term treatment with Obex®, combined with lifestyle changes, appears to provide additional health benefits to subjects with overweight and obesity. Obex® represents a new alternative therapy in patients with overweight, obesity, prediabetes and other diseases characterised by insulin resistance.

## Data Availability

The datasets used and/or analysed during the current study available from the corresponding author on reasonable request.

## References

[1] Inoue Y, Qin B, Poti J, Sokol R, Gordon-Larsen P (2018). Epidemiology of Obesity in Adults: Latest Trends. Curr Obes Rep.

[2] Okunogbe A, Nugent R, Spencer G, Ralston J, Wilding J. Economic impacts of overweight and obesity: current and future estimates for eight countries. BMJ Glob Health. 2021;6(10):e006351. 10.1136/bmjgh-2021-006351. 10.1136/bmjgh-2021-006351 PMC8487190 34737167

[3] De Lorenzo A, Gratteri S, Gualtieri P, Cammarano A, Bertucci P, Di Renzo L. Why primary obesity is a disease? J Transl Med. 2019;17(1):169. 10.1186/s12967-019-1919-y. 10.1186/s12967-019-1919-y PMC6530037 31118060

[4] Global Burden of Metabolic Risk Factors for Chronic Diseases Collaboration (BMI Mediated Effects), Lu Y, Hajifathalian K, Ezzati M, Woodward M, Rimm EB, et al. Metabolic mediators of the effects of body-mass index, overweight, and obesity on coronary heart disease and stroke: a pooled analysis of 97 prospective cohorts with 1.8 million participants. Lancet. 2014;383(9921):970–83. 10.1016/S0140-6736(13)61836-X PMC3959199 24269108

[5] Jensen MD, Ryan DH, Apovian CM, Ard JD, Comuzzie AG, Donato KA (2014). 2013 AHA/ACC/TOS guideline for the management of overweight and obesity in adults: a report of the American College of Cardiology/American Heart Association Task Force on Practice Guidelines and the Obesity Society. Circulation.

[6] Burgio E, Lopomo A, Migliore L (2015). Obesity and diabetes: from genetics to epigenetics. Mol Biol Rep..

[7] Bray GA, Heisel WE, Afshin A, Jensen MD, Dietz WH, Long M, Kushner RF, Daniels SR, Wadden TA, Tsai AG, Hu FB, Jakicic JM, Ryan DH, Wolfe BM, Inge TH (2018). The Science of Obesity Management: An Endocrine Society Scientific Statement. Endocr Rev.

[8] Ismail L, Materwala H, Al Kaabi J (2021). Association of risk factors with type 2 diabetes: A systematic review. Comput Struct Biotechnol J..

[9] Miao Z, Alvarez M, Ko A, Bhagat Y, Rahmani E, Jew B, Heinonen S, et al. The causal effect of obesity on prediabetes and insulin resistance reveals the important role of adipose tissue in insulin resistance. PLoS Genet. 2020;16(9):e1009018. 10.1371/journal.pgen.1009018. 10.1371/journal.pgen.1009018 PMC7515203 32925908

[10] Kamel EG, McNeill G, Van Wijk MC (2000). Usefulness of anthropometry and DXA in predicting intra-abdominal fat in obese men and women. Obes Res..

[11] Reis JP, Macera CA, Araneta MR, Lindsay SP, Marshall SJ, Wingard DL (2009). Comparison of overall obesity and body fat distribution in predicting risk of mortality. Obesity (Silver Spring)..

[12] Díaz ME, Jiménez S, García RG, Bonet M, Wong I (2009). Overweight, obesity, central adiposity and associated chronic diseases in cuban adults. MEDICC Rev..

[13] Castellanos-González M, Benet-Rodríguez M, Morejón-Giraldoni A, Colls-Cañizares Y. Obesidad abdominal, parámetro antropométrico predictivo de alteraciones del metabolismo. Revista Finlay [revista en Internet]. 2011;1(2). [cited 2023 Jan 27]. Available in: https://revfinlay.sld.cu/index.php/finlay/article/view/40.

[14] Liu B, Du Y, Wu Y, Snetselaar LG, Wallace RB, Bao W. Trends in obesity and adiposity measures by race or ethnicity among adults in the United States 2011–18: population based study. BMJ. 2021;372:n365. 10.1136/bmj.n365. 10.1136/bmj.n365 PMC7961695 33727242

[15] Jiménez-Acosta S, Rodríguez-Suárez A, Díaz-Sánchez M. La obesidad en Cuba. Una mirada a su evolución en diferentes grupos poblacionales. Revista Cubana de Alimentación y Nutrición [Internet]. 2013;23(2). [cited 27 Jan 2023]. Available in: https://revalnutricion.sld.cu/index.php/rcan/article/view/299.

[16] GBD 2015 Obesity Collaborators; Afshin A, Forouzanfar MH, Reitsma MB, Sur P, Estep K, et al. Health Effects of Overweight and Obesity in 195 Countries over 25 Years. N Engl J Med. 2017;377(1):13–27. 10.1056/NEJMoa1614362 PMC5477817 28604169

[17] Serra-Majem L, Bautista-Castaño I. Etiology of obesity: two "key issues" and other emerging factors. Nutr Hosp. 2013;28 (Suppl 5):32–43. 10.3305/nh.2013.28.sup5.6916 24010742

[18] Speakman JR, O'Rahilly S (2012). Fat: an evolving issue. Dis Model Mech..

[19] Webber L, Kilpi F, Marsh T, Rtveladze K, Brown M, McPherson K. High rates of obesity and non-communicable diseases predicted across Latin America. PLoS One. 2012;7(8):e39589. 10.1371/journal.pone.0039589. 10.1371/journal.pone.0039589 PMC3418261 22912663

[20] Newgard CB, An J, Bain JR, Muehlbauer MJ, Stevens RD, Lien LF (2009). A branched-chain amino acid-related metabolic signature that differentiates obese and lean humans and contributes to insulin resistance. Cell Metab..

[21] Uusitupa M, Khan TA, Viguiliouk E, Kahleova H, Rivellese AA, Hermansen K, Pfeiffer A, et al. Prevention of Type 2 Diabetes by Lifestyle Changes: A Systematic Review and Meta-Analysis. Nutrients. 2019;11(11):2611. 10.3390/nu11112611. 10.3390/nu11112611 PMC6893436 31683759

[22] La Sala L, Pontiroli AE. Prevention of Diabetes and Cardiovascular Disease in Obesity. Int J Mol Sci. 2020;21(21):8178. 10.3390/ijms21218178. 10.3390/ijms21218178 PMC7663329 33142938

[23] Müller TD, Blüher M, Tschöp MH, DiMarchi RD (2022). Anti-obesity drug discovery: advances and challenges. Nat Rev Drug Discov..

[24] Gadde KM, Apolzan JW, Berthoud HR (2018). Pharmacotherapy for patients with obesity. Clin Chem.

[25] Lee CMY, Woodward M, Pandeya N, Adams R, Barrett-Connor E, Boyko EJ (2017). Comparison of relationships between four common anthropometric measures and incident diabetes. Diabetes Res Clin Pract..

[26] Scheen AJ, Van Gaal LF (2014). Combating the dual burden: therapeutic targeting of common pathways in obesity and type 2 diabetes. Lancet Diabetes Endocrinol..

[27] Narayanaswami V, Dwoskin LP (2017). Obesity: Current and potential pharmacotherapeutics and targets. Pharmacol Ther..

[28] Udani JK, Singh BB, Singh VJ, Barrett ML. Effects of Açai (Euterpe oleracea Mart.) berry preparation on metabolic parameters in a healthy overweight population: a pilot study. Nutr J. 2011;10:45. 10.1186/1475-2891-10-45. 10.1186/1475-2891-10-45 PMC3118329 21569436

[29] Santos IB, de Bem GF, da Costa CA, de Carvalho LCRM, de Medeiros AF, Silva DLB (2020). Açaí seed extract prevents the renin-angiotensin system activation, oxidative stress and inflammation in white adipose tissue of high-fat diet-fed mice. Nutr Res..

[30] Wang L, Yang H, Huang H, Zhang C, Zuo HX, Xu P (2019). Inulin-type fructans supplementation improves glycemic control for the prediabetes and type 2 diabetes populations: results from a GRADE-assessed systematic review and dose-response meta-analysis of 33 randomized controlled trials. J Transl Med..

[31] Vitalone A, Di Sotto A, Mammola CL, Heyn R, Miglietta S, Mariani P, Sciubba F, Passarelli F, Nativio P, Mazzanti G (2017). Phytochemical analysis and effects on ingestive behaviour of a Caralluma fimbriata extract. Food Chem Toxicol..

[32] Watanabe M, Risi R, Masi D, Caputi A, Balena A, Rossini G, et al. Current Evidence to Propose Different Food Supplements for Weight Loss: A Comprehensive Review. Nutrients. 2020;12(9):2873. 10.3390/nu12092873. 10.3390/nu12092873 PMC7551574 32962190

[33] Rao A, Briskey D, Dos Reis C, Mallard AR. The effect of an orally-dosed Caralluma Fimbriata extract on appetite control and body composition in overweight adults. Sci Rep. 2021;11(1):6791. 10.1038/s41598-021-86108-2. 10.1038/s41598-021-86108-2 PMC7991653 33762661

[34] Anwar R, Rabail R, Rakha A, Bryla M, Roszko M, Aadil RM, et al. Delving the Role of Caralluma fimbriata: An Edible Wild Plant to Mitigate the Biomarkers of Metabolic Syndrome. Oxid Med Cell Longev. 2022. 10.1155/2022/5720372. 10.1155/2022/5720372 PMC9236770 35770046

[35] Durazzo A, Lucarini M, Nazhand A, Souto SB, Silva AM, Severino P, et al. The nutraceutical value of carnitine and its use in dietary supplements. Molecules. 2020;25(9):2127. 10.3390/molecules25092127. 10.3390/molecules25092127 PMC7249051 32370025

[36] Savic D, Ball V, Curtis MK, Sousa Fialho MDL, Timm KN, et al. L-Carnitine Stimulates In Vivo Carbohydrate Metabolism in the Type 1 Diabetic Heart as Demonstrated by Hyperpolarized MRI. Metabolites. 2021;11(3):191. 10.3390/metabo11030191. 10.3390/metabo11030191 PMC8004902 33806953

[37] Du X, Shi L, Gao H, Fu X, Zhang X, Zhang Y, et al. The effect of zinc supplementation in pre-diabetes: A protocol for systematic review and meta-analysis. Medicine (Baltimore). 2019;98(27):e16259. 10.1097/MD.0000000000016259. 10.1097/MD.0000000000016259 PMC6635284 31277146

[38] Shi Z (2020). Anti-Obesity Effects of α-Amylase Inhibitor Enriched-Extract from White Common Beans (Phaseolus Vulgaris L.) Associated with the Modulation of Gut Microbiota Composition in High-Fat Diet-Induced Obese Rats. Food Funct.

[39] Avagliano C, De Caro C, Cuozzo M, Liguori FM, La Rana G, Micheli L, et al. Phaseolus vulgaris extract ameliorates high-fat diet-induced colonic barrier dysfunction and inflammation in mice by regulating peroxisome proliferator-activated receptor expression and butyrate levels. Front Pharmacol. 2022;13:930832. 10.3389/fphar.2022.930832. 10.3389/fphar.2022.930832 PMC9403263 36034787

[40] Sid V, Shang Y, Siow YL, Hewage SM, House JD, O K (2018). Folic Acid Supplementation Attenuates Chronic Hepatic Inflammation in High-Fat Diet Fed Mice. Lipids.

[41] Borah PK, Sarkar A, Duary RK (2019). Water-soluble vitamins for controlling starch digestion: Conformational scrambling and inhibition mechanism of human pancreatic α-amylase by ascorbic acid and folic acid. Food Chem.

[42] Asbaghi O, Ashtary-Larky D, Bagheri R, Moosavian SP, Olyaei HP, Nazarian B, et al. Folic Acid Supplementation Improves Glycemic Control for Diabetes Prevention and Management: A Systematic Review and Dose-Response Meta-Analysis of Randomized Controlled Trials. Nutrients. 2021;13(7):2355. 10.3390/nu13072355. 10.3390/nu13072355 PMC8308657 34371867

[43] Navik U, Rawat K, Tikoo K. L-Methionine prevents β-cell damage by modulating the expression of Arx, MafA and regulation of FOXO1 in type 1 diabetic rats. Acta Histochem. 2022;124(1):151820. 10.1016/j.acthis.2021.151820. 10.1016/j.acthis.2021.151820 34871948

[44] Hu S, Han M, Rezaei A, Li D, Wu G, Ma X (2017). L-Arginine Modulates Glucose and Lipid Metabolism in Obesity and Diabetes. Curr Protein Pept Sci.

[45] Monti LD, Galluccio E, Villa V, Fontana B, Spadoni S, Piatti PM (2018). Decreased diabetes risk over 9 year after 18-month oral L-arginine treatment in middle-aged subjects with impaired glucose tolerance and metabolic syndrome (extension evaluation of L-arginine study). Eur J Nutr.

[46] Medialdea L, Bodas I, Carmenate MM, Del Valle A, Marrodán MD, Prado C (2015). Effectivenes of dietary supplementation with Caralluma fimbriata in metabolic syndrome reduction during climacteric period. Nutr clín diet hosp.

[47] Carreira MC, Andrade S, Gonzalez-Izquierdo A, Amil M, Folgueira C, Monteiro MP, et al. Anti-obesity activity of OBEX is regulated by activation of thermogenesis and decreasing adiposity gain. Sci Rep. 2018;8(1):17155. 10.1038/s41598-018-34840-7. 10.1038/s41598-018-34840-7 PMC6249269 30464239

[48] Cabrera-Rode E, Rodríguez-Acosta J, Álvarez-Álvarez A, Echevarria-Valdés R, Reyes-Rodríguez A, Cubas-Dueñas I, Turcios-Tristá SE, Díaz-Díaz O (2017). Effects of Obex® in overweight and obese subjects with and without impaired fasting glucose: a pilot study. J Diet Suppl.

[49] Cabrera-Rode E, Orlandi N, Padrón Y, Arranz C, Olano R, Machado M, Hernández-Yero A, Calderín R, Domínguez E (2013). Effect of Diamel in patients with metabolic syndrome: a randomized double-blind placebo-controlled study. J Diabetes.

[50] Craig CL, Marshall AL, Sjostrom M, Bauman AE, Booth ML, Ainsworth BE (2003). International physical activity questionnaire: 12-country reliability and validity. Med Sci Sports Exerc.

[51] Rodgers M, Migdal AL, Rodríguez TG, Chen ZZ, Nath AK, Gerszten RE, et al. Weight loss outcomes among early high responders to exenatide treatment: a randomized, placebo controlled study in overweight and obese women. Front Endocrinol (Lausanne). 2021;12:742873. 10.3389/fendo.2021.742873. 10.3389/fendo.2021.742873 PMC8635796 34867786

[52] Valdez R (1991). A simple model-based index of abdominal adiposity. J Clin Epidemiol..

[53] Kundu PK, Cohen IM, Dowling DR. Fluid mechanics. Waltham: Academic Press; 2012.

[54] Muntner P, Einhorn PT, Cushman WC, Whelton PK, Bello NA, Drawz PE (2019). Blood pressure assessment in adults in clinical practice and clinic-based research: jacc scientific expert panel. J Am Coll Cardiol..

[55] Matthews DR, Hosker JP, Rudenski AS, Naylor BA, Treacher DF, Turner RC (1985). Homeostasis model assessment: insulin resistance and beta-cell function from fasting plasma glucose and insulin concentrations in man. Diabetologia..

[56] Arranz C, González RM, Álvarez A, Rodríguez B, Reyes A (2010). Reference criteria for insulin secretion indicators and of the lipid parameters in a hospital mixed population. Rev Cubana Endocrinol..

[57] Singh B, Saxena A (2010). Surrogate markers of insulin resistance: a review. World J Diabetes..

[58] Fujioka K, O’Neil PM, Davies M, Greenway F, Lau CW, Claudius B, et al. Early weight loss with Liraglutide 3.0 Mg predicts 1-year weight loss and is associated with improvements in clinical markers. Obesity. 2016;24(11):2278–88. 10.1002/oby.21629. 10.1002/oby.21629 PMC5129670 27804269

[59] Saura J, Isidro F, Heredia JR, Segarra V (2014). Evidencias científicas sobre la eficacia y seguridad de la dieta proteinada: dieta proteinada y ejercicio físico. Rev Andal Med Deporte.

[60] Brosens C (2009). Barreras en la adherencia al tratamiento de la obesidad. Evid Actual Práct Ambul.

[61] Rubio Herrera MA, Moreno Lopera C (2005). Medicina basada en la evidencia: nutrición en la obesidad. Endocrinol Nutr.

[62] Pi-Sunyer X, Astrup A, Fujioka K, Greenway F, Halpern A, Krempf M (2015). A Randomized, Controlled Trial of 3.0Mg of Liraglutide in Weight Management. N Engl J Med.

[63] Dushay J, Gao C, Gopalakrishnan GS, Crawley M, Mitten EK, Wilker E (2012). Short-term Exenatide treatment leads to significant weight loss in a subset of obese women without diabetes. Diabetes Care.

[64] O’Neil P, Birkenfeld AL, McGowan B, Mosenzon O, Pedersen SD, Wharton S (2018). Efficacy and Safety of Semaglutide Compared With Liraglutide and Placebo for Weight Loss in Patients With Obesity: A Randomised, Double-Blind, Placebo and Active Controlled, Dose-Ranging, Phase 2 Trial. Lancet.

[65] Baillot A, Romain AJ, Boisvert-Vigneault K, Audet M, Baillargeon JP, Dionne IJ, et al. Effects of lifestyle interventions that include a physical activity component in class II and III obese individuals: a systematic review and meta-analysis. PLoS One. 2015;10(4):e0119017. 10.1371/journal.pone.0119017. 10.1371/journal.pone.0119017 PMC4382170 25830342

[66] de Almeida Magalhães TSS, de Oliveira Macedo PC, Converti A, Neves de Lima ÁA. The Use of Euterpe oleracea Mart. As a New Perspective for Disease Treatment and Prevention. Biomolecules. 2020;10(6):813. 10.3390/biom10060813. 10.3390/biom10060813 PMC7356995 32466439

[67] Li L, Li P, Xu L (2021). Assessing the effects of inulin-type fructan intake on body weight, blood glucose, and lipid profile: a systematic review and meta-analysis of randomized controlled trials. Food Sci Nutr.

[68] Bystad M, Bystad C, Wynn R (2015). How can placebo effects best be applied in clinical practice? A narrative review. Psychol Res Behav Manag..

[69] Enck P, Klosterhalfen S (2019). Placebos and the Placebo Effect in Drug Trials. Handb Exp Pharmacol..

[70] Meissner K, Distel H, Mitzdorf U (2007). Evidence for placebo effects on physical but not on biochemical outcome parameters: a review of clinical trials. BMC Med..

[71] Chambers ES, Byrne CS, Morrison DJ, Murphy KG, Preston T, Tedford C (2019). Dietary supplementation with inulin-propionate ester or inulin improves insulin sensitivity in adults with overweight and obesity with distinct effects on the gut microbiota, plasma metabolome and systemic inflammatory responses: a randomised cross-over trial. Gut.

[72] Yu P, Huang L, Wang Z, Meng X, Yu X (2021). The Association of Serum Uric Acid with Beta-Cell Function and Insulin Resistance in Nondiabetic Individuals: A Cross-Sectional Study. Diabetes Metab Syndr Obes..

[73] Zhi L, Yuzhang Z, Tianliang H, Hisatome I, Yamamoto T, Jidong C. High Uric Acid Induces Insulin Resistance in Cardiomyocytes In Vitro and In Vivo. PLoS One. 2016;11(2):e0147737. 10.1371/journal.pone.0147737. 10.1371/journal.pone.0147737 PMC4737875 26836389

[74] Gong M, Wen S, Nguyen T, Wang C, Jin J, Zhou L (2020). Converging Relationships of Obesity and Hyperuricemia with Special Reference to Metabolic Disorders and Plausible Therapeutic Implications. Diabetes Metab Syndr Obes..

[75] Mwasongwe SE, Fülöp T, Katz R, Musani SK, Sims M, Correa A, Flessner MF, Young BA (2018). Relation of uric acid level to rapid kidney function decline and development of kidney disease: The Jackson Heart Study. J Clin Hypertens (Greenwich).

[76] Usman M, Zhang C, Patil PJ, Mehmood A, Li X, Bilal M, et al. Potential applications of hydrophobically modified inulin as an active ingredient in functional foods and drugs - A review. Carbohydr Polym. 2021;252:117176. 10.1016/j.carbpol.2020.117176. 10.1016/j.carbpol.2020.117176 PMC7536552 33183623

[77] Hughes RL, Alvarado DA, Swanson KS, Holscher HD (2021). The Prebiotic Potential of Inulin-type Fructans: A Systematic Review. Adv Nutr..

[78] Batterham M, Tapsell LC, Charlton KE (2016). Predicting dropout in dietary weight loss trials using demographic and early weight change characteristics: implications for trial design. Obes Res Clin Pract.

[79] Yackobovitch-Gavan M, Steinberg DM, Endevelt R, Benyamini Y. Factors associated with dropout in a group weight-loss programme: a longitudinal investigation. J Hum Nutr Diet. 2015;28 Suppl 2:33–40. 10.1111/jhn.12220. 10.1111/jhn.12220 24528102

